# SSFVEP as a potential electrophysiological examination for evaluating visual function of fundus diseases with vitreous hemorrhages: a clinical study

**DOI:** 10.1038/s41598-023-47714-4

**Published:** 2024-01-29

**Authors:** Weiming Yan, Qiurui He, Meizhu Chen, Sheng Zhang, Tao Chen, Lei Zhang, Haiyan Wang

**Affiliations:** 1 The Shaanxi Eye Hospital, Xi’an People’s Hospital (Xi’an Fourth Hospital), Xi’an, China; 2 The Third Hospital of Zhangzhou, Zhangzhou, China; 3 Fuzong Clinical Medical College of Fujian Medical University, Dongfang Hopsital Affiliated to Xiamen University, Fuzhou, China; 4 Center of Clinical Aerospace Medicine, Air Force Military Medical University, Xi′an, China

**Keywords:** Neuroscience, Medical research

## Abstract

To investigate the sensitivity and potential application of steady-state flash visual evoked potentials (SSFVEP) in assessing the visual function of fundus diseases with vitreous hemorrhage. 18 patients diagnosed with monocular vitreous hemorrhages in the fundus were examined the flash visual evoked potentials (FVEP) and SSFVEP in both eyes. The difference in the P2-wave amplitude of FVEP and the average amplitude of SSFVEP waveform between the diseased eyes and those without vitreous hemorrhage were statistically compared. There was no significant difference in the waveform of FVEP between both eyes. The amplitude of P2-wave from FVEP of the diseased eye was slightly lower than that without vitreous hemorrhage. However, the difference was not statistically significant (*P* = 0.111). The waveform of SSFVEP in the eye without vitreous hemorrhage showed a towering shape, while that of the diseased eye was flat. The average amplitude of SSFVEP in the diseased eye was statistically lower than that without vitreous hemorrhage (*P* = 0.036). The difference ratio of SSFVEP amplitude between both eyes was significantly greater than that of FVEP amplitude (*P* = 0.028). In some fundus diseases with vitreous hemorrhage, SSFVEP had a higher sensitivity than FVEP, providing a novel potential application for visual function assessment.

## Introduction

Visual electrophysiological examinations, widely utilized in cases where a diagnosis is elusive, have been employed in clinical settings for centuries^1, 2^. The visual evoked potentials (VEP) belongs to one of the commonly used visual electrophysiological methods and includes pattern visual evoked potentials (PVEP) and flash visual evoked potentials (FVEP)^3^. VEP is often used to predict and evaluate visual function in ophthalmic diseases, especially when opacities in the ocular media (such as cataracts and vitreous hemorrhage) affect morphological examinations of the fundus, such as fundus photography and optical coherence tomography (OCT)^4, 5^. It can be used to evaluate the functional integrity of the visual system, encompassing the ocular media, retina, optic nerve, and visual cortex. FVEP, being more variable than PVEP in different subjects, generally exhibits similarity between both eyes within an individual subject^6^. FVEP proves useful in assessing visual function among patients with ocular media opacities that would impede the reliable use of PVEP^7^.

Steady-state flash VEP (SSFVEP) is a type of VEP, which stimulates the eyes with fixed-frequency flashes. The flashing stimulus frequency for SSFVEP is usually more than 4 Hz, which is higher than that of the FVEP^8^. The stimulating of SSFVP could cause the visual cortex in the brain to generate periodic stimulation rhythms, and the corresponding evoked potentials are recorded at the occipital lobe^9, 10^. Previously, a few studies applied SSFVEP to predict postoperative visual function in some cataract patients and showed promising results^11^. However, the application of SSFVEP in assessing the visual function has not been widely recognized.

In this study, FVEP and SSFVEP, the two visual electrophysiological examinations, were performed on patients with unilateral vitreous hemorrhage collected at our hospital. The different sensitivity of parameters derived from FVEP and SSFVEP in eyes with or without vitreous hemorrhage were further compared and analyzed. Our work aimed to explore the potential role of SSFVEP in evaluating visual function in ocular diseases.

## Subjects and methods

### General information of the subjects

**Figure 1:**
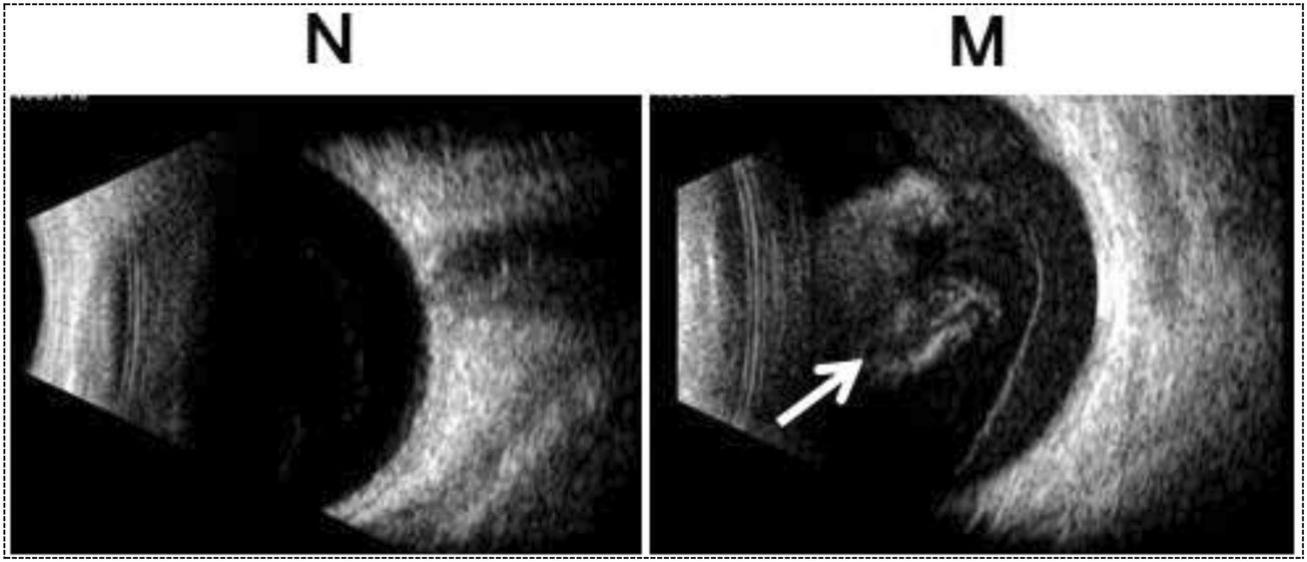
Typical pictures of the B-scan ultrasonography in eyes without vitreous hemorrhage and eyes with vitreous hemorrhage. An obvious vitreous hemorrhage was shown in the typical pictures of the B-scan ultrasonography in eyes with vitreous hemorrhage, compared to that of the normal eye. N: eyes without vitreous hemorrhage; M: eyes with vitreous hemorrhage; The white arrow: the vitreous hemorrhage.

A retrospective analysis was conducted on 18 patients with unilateral vitreous hemorrhage who were treated at our hospital from May 1, 2020 to August 31, 2021. We had access to information that could identify individual participants during or after data collection. Among them, there were 10 male and 8 female patients, aged between 38 and 66 years old, with an average age of 52 ± 0.7 years. None of them had a history of trauma. According to clinical diagnosis (based on ocular ultrasound (Fig. 1), fundus fluorescein angiography, optical coherence tomography, and other relevant examinations), the patients were classified as follows: vitreous hemorrhage with diabetic retinopathy (9 eyes), vitreous hemorrhage with retinal vein occlusion (5 eyes), and vitreous hemorrhage with polypoidal choroidal vasculopathy (4 eyes). Among them, 12 eyes were right eyes and 6 eyes were left eyes, and the visual acuity of all eyes was less than 0.02 in *logMAR*. There was no history of previous ocular trauma. No vitreous hemorrhage was found in the normal eye, with a naked eye central vision or corrected visual acuity more than 0.8 in *logMAR*. This study was approved by the Ethics Committee of Fuzong Clinical Medical College of Fujian Medical University with the clinical trial registration number of No. #2020002182 at January 1^st^, 2020. Written informed consent was obtained from all the patients. Besides, all methods in our study were performed in accordance with the relevant guidelines and regulations.

### Methods

#### Equipment of the electrophysiological examination

The RETI port/scan 21 visual electrophysiological examination system from Germany's Roland Company was used, in accordance with the standards of the International Society for Clinical Electrophysiology of Vision (ISCEV)^12^. Visual electrophysiological testing (FVEP and SSFVEP) was performed on the patients using a Ganzfeld stimulator.

#### Parameters of the examinations

Flash visual evoked potentials (FVEP) were recorded according to the ISCEV standards^8, 13^. The specific parameters were as follows: a Ganzfeld stimulator was used, the flash intensity was 0.5 cd·s/m^2^, the stimulation frequency was 1 Hz, the number of stimulation superimpositions was 60, the sampling frequency was 1.0 Hz, the amplification factor was 80,000 times, the passband was 1–50 Hz, and the recording time was 200 ms.

Steady-state flash visual evoked potentials (SSFVEP) were recorded according to the ISCEV standards^13^. The same Ganzfeld stimulator used for FVEP was used, and the specific parameters were as follows: the flash intensity was 0.5 cd·s/m^2^, the stimulation frequency was 12 Hz, the number of stimulation superimpositions was 60, the sampling frequency was 1.0 Hz, the amplification factor was 80,000 times, the passband was 1–50 Hz, and the recording time was 200 ms.

#### Examination procedure for electrophysiology detection

1. Skin cleaning: Clean the skin at the mid-frontal area below the hairline, one side of the earlobe, and 1.5–2.0 cm above the occipital protuberance with a skin cleanser.
2. Electrodes placement: Use disc-shaped skin electrodes, apply conductive gel to the electrode surface, and fix them on the cleaned skin. According to the ISCEV standard, the FVEP and SSFVEP recording electrodes are placed 2 cm above the occipital protuberance, with the reference electrode placed on the mid-frontal skin, and the ground electrode placed on the earlobe skin.
3. Impedance checking: Check the electrical impedance at the electrode–skin contact points, ensuring it is less than 5 kΩ. If the impedance at any electrode exceeds 5 kΩ, clean the corresponding skin area again with a skin cleanser.
4. FVEP examination: After placing and confirming the appropriate electrode impedance, cover the affected eye (with vitreous hemorrhage) with a black eye patch. Rest the chin on the chin rest of the Ganzfeld stimulator, support the forehead against it, and ask the patient to fixate on the red dot inside the stimulator. Stimulate the eye according to the parameters mentioned above, overlaying 60 trials. Stop the detection once the FVEP waveform becomes stable. After closing both eyes for one minute, repeat the FVEP detection to ensure waveform consistency. Change the eye patch to cover the unaffected eye and repeat the same procedure to detect FVEP for the affected eye.
5. SSFVEP examination: After placing and confirming the appropriate electrode impedance using the aforementioned method, use the Ganzfeld stimulator to separately detect the SSFVEP waveform for both eyes. The stimulation frequency is set at 12 Hz. The remaining steps of the detection procedure are the same as for FVEP.

#### Parameters recording

Record the P2-wave amplitude in FVEP waveforms (in μV) for both eyes with and without vitreous hemorrhage, and record the average amplitude in SSFVEP waveforms (in μV) for both eyes. For the SSFVEP waveforms, we performed the Fourier analysis method to reduce the influence of background noise and to facilitate the statistical analysis of the amplitudes in the waveforms.

### Statistical analysis

The data were processed using SPSS 16.0 statistical software. The P2-wave amplitude in FVEP, the average amplitude from SSVEP, and other indicators were presented as mean ± standard error (± SE). The paired t-test was applied to compare the amplitude of P2-wave in FVEP, and the average amplitude of SSFVEP between eyes with and without vitreous hemorrhage. Furthermore, the difference of the amplitude ratio of eyes with vitreous hemorrhage to eyes without hemorrhage between SSVEP and FVEP was compared by the Wicoxon test. A *P* value less than 0.05 was set as a statistical difference.

### Ethical approval

This study was reviewed and approved by the Ethics Committee of Fuzong Clinical Medical College of Fujian Medical University with the clinical trial registration number of No. #2020002182 at January 1st, 2020. Besides, all methods were performed in accordance with the relevant guidelines and regulations. All patients provided informed consent to participate in the study and provided informed consent for the publication of their anonymised case details and images.

## Results

As showed in the examination of the B-scan ultrasonography, there was an obvious sign of hemorrhage in the vitreous body in the typical pictures of the B-scan ultrasonography in eyes with vitreous hemorrhage. Some parts of hemorrhage were in higher density than the other parts. Meanwhile, the vitreous body was relatively clear in that of the normal eye. No other opacities were found insides the vitreous body (Fig. 1).

**Figure 2:**
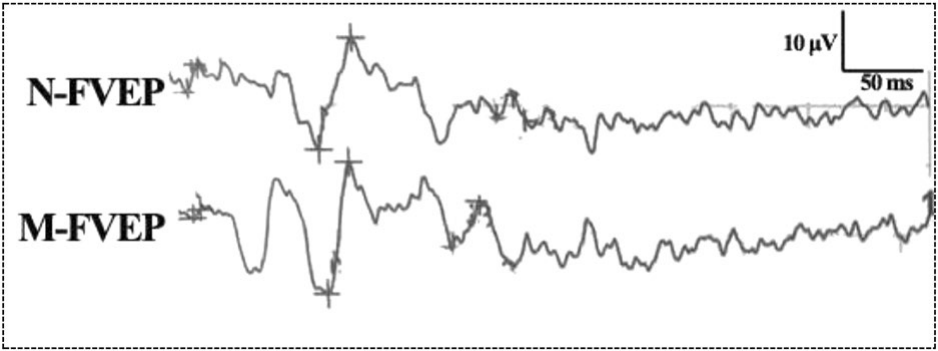
Typical waveforms of flash visual evoked potentials (FVEP) in eyes without vitreous hemorrhage and eyes with vitreous hemorrhage. Both eyes showed a visible P2-wave in the FVEP examination. No significant differences in the FVEP waveforms between eyes with and without vitreous hemorrhage were found. FVEP: flash visual evoked potentials; N-FVEP: the FVEP waveform in eyes without vitreous hemorrhage; M-FVEP: the FVEP waveform in eyes with vitreous hemorrhage; the upper symbol of “+”: the position of P2-wave.

**Figure 3:**
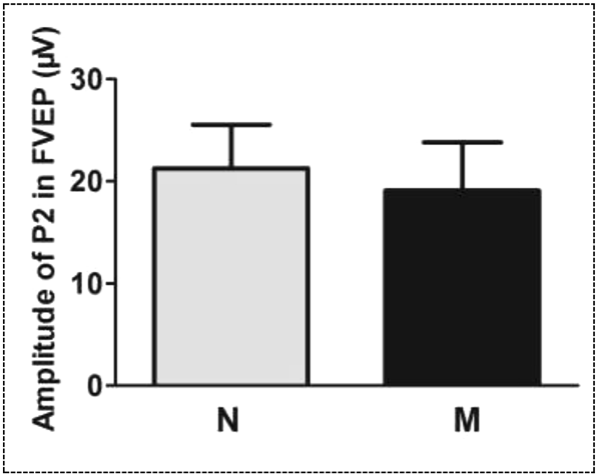
The statistical graph of P2-wave amplitudes in flash visual evoked potentials (FVEP) between eyes without vitreous hemorrhage and eyes with vitreous hemorrhage. No statistical significance was found in the difference in P2-wave amplitude between the eyes with or without vitreous hemorrhage. N: eyes without vitreous hemorrhage; M: eyes with vitreous hemorrhage; FVEP: flash visual evoked potentials; P2: the P2-wave of the FVEP.

In the examination of FVEP, an obvious waveform was elicited from both eyes. There were no significant differences in the FVEP waveforms between eyes with and without vitreous hemorrhage. Both eyes showed a visible P2-wave, the most obvious peak of the potential, in the FVEP (Fig. 2). In details, the amplitude of the P2-wave of FVEP in eyes with vitreous hemorrhage was 19.06 ± 4.75 μV, while in eyes without vitreous hemorrhage, the data was 21.26 ± 4.28 μV. The difference in P2-wave amplitude between the two eyes was not statistically significant (t = 2.037, *P* = 0.111) (Fig. 3).

**Figure 4:**
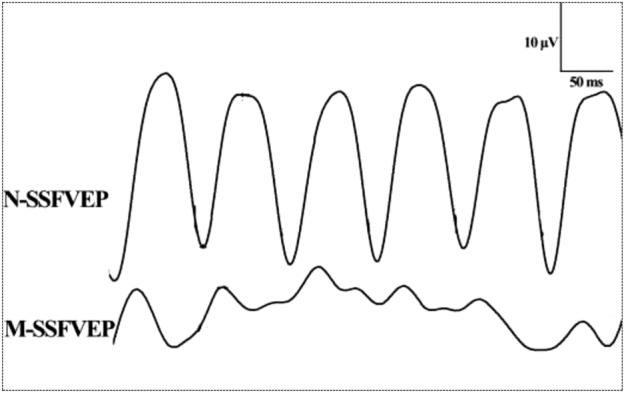
Typical waveform of steady-state flash visual evoked potentials (SSFVEP) in eyes without vitreous hemorrhage and eyes with vitreous hemorrhage after the Fourier processing. The SSVEP waveforms in eyes without vitreous hemorrhage appeared as high and steep sinusoidal waves, while the waveforms were lower and flatter in eyes with vitreous hemorrhage. SSFVEP: steady-state flash visual evoked potentials; N-SSFVEP: the SSFVEP waveform in eyes without vitreous hemorrhage; M-SSFVEP: the SSFVEP waveform in eyes with vitreous hemorrhage.

In the examination of SSFVEP, the SSVEP waveforms in eyes without vitreous hemorrhage appeared obvious and high. The waveform showed a steep sinusoidal-like shape with a series of steady regular waves. Besides, small peaks were found on each sinusoidal-like wave, which were removed after the Fourier processing. On the contrary, in eyes with vitreous hemorrhage, the SSFVEP waveforms were lower and flatter. No obvious waveforms of sinusoidal-like shape were elicited from the stimulation (Fig. 4).

**Figure 5:**
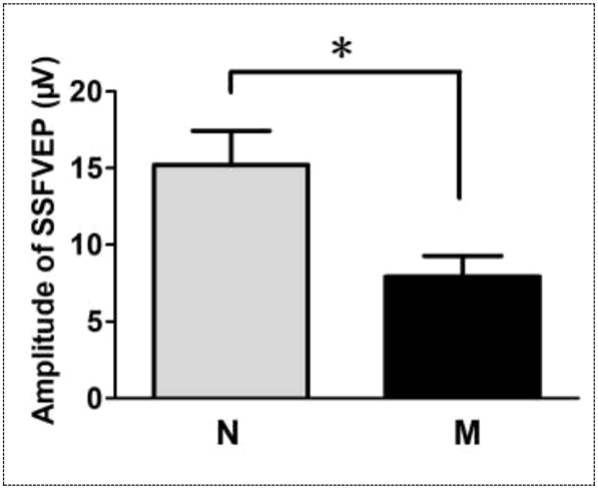
The statistical graph of the average amplitudes in the steady-state flash visual evoked potentials (SSFVEP) between eyes without vitreous hemorrhage and eyes with vitreous hemorrhage. A statistical significance was found in the difference in the average amplitudes of SSFVEP between the eyes with or without vitreous hemorrhage. N: eyes without vitreous hemorrhage; M: eyes with vitreous hemorrhage; SSFVEP: steady-state flash visual evoked potentials; *: *P* < 0.05.

For the statistical analysis afer the Fourier processing, the average amplitude of the SSFVEP wave in eyes without vitreous hemorrhage was 15.20 ± 2.23 μV, while in eyes with vitreous hemorrhage, it was 7.92 ± 1.35 μV. The amplitude of the SSFVEP wave in eyes with vitreous hemorrhage was significantly lower compared to eyes without vitreous hemorrhage, and the difference in amplitude between the two eyes was statistically significant (t = 2.86, *P* = 0.036) (Fig. 5).

**Figure 6:**
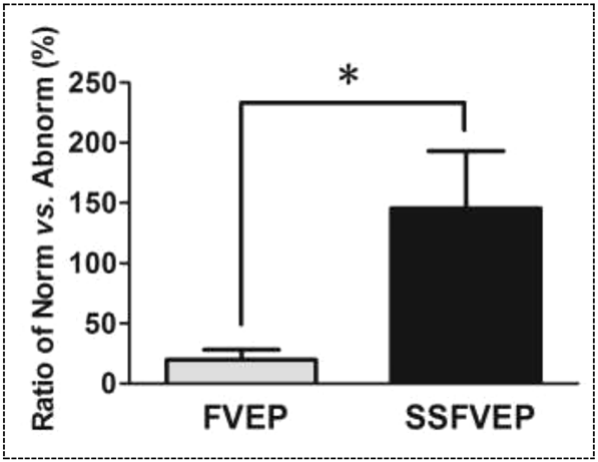
Comparative statistical graph of the difference ratios of the amplitudes from eyes with and without vitreous hemorrhage in flash visual evoked potentials (FVEP) and steady-state flash visual evoked potentials (SSFVEP). There was a statistical significance in the difference ratios of the amplitudes from eyes with and without vitreous hemorrhage between FVEP and SSFVEP examination. FVEP: flash visual evoked potentials; SSFVEP: steady-state flash visual evoked potentials; Norm: the amplitudes in eyes without vitreous hemorrhage; Abnorm: the amplitudes in eyes with vitreous hemorrhage; *: *P* < 0.05.

In greater details, the average ratio of the amplitude of the P2-wave of FVEP in eyes with vitreous hemorrhage compared to eyes without vitreous hemorrhage was 19.77 ± 8.16%. Meanwhile, the average ratio of the amplitude of the SSFVEP wave in eyes with vitreous hemorrhage compared to eyes without vitreous hemorrhage was 158.55 ± 56.27%. The difference in the amplitude ratio of SSVEP between the two eyes was significantly greater than that of FVEP, and the difference was statistically significant (z = − 2.201, *P* = 0.028) (Fig. 6).

## Discussion

Visual electrophysiology plays an irreplaceable role as an objective examination indicator in ophthalmic clinical applications. VEP reflects the conduction function of the entire visual pathway from visual stimulation through the ocular media to the retina, then the optic nerve, and finally to the cortical visual center. Conventional PVEP and FVEP can be used for visual function evaluation, which can reflect the light sensitivity of the entire retinal optic nerve, including coarse incoming fibers from the peripheral retinal area and fine incoming fibers from the macula. The application of PVEP and FVEP in various studies, including optic nerve diseases, has been widely reported^14, 15^. Furthermore, when accompanied by opacities in the ocular media, the role and significance of FVEP in the diagnosis and evaluation of visual function can be further demonstrated^16, 17^.

FVEP belongs to transient FVEP, with a stimulation frequency of 1 Hz. Its individual variation is often significant, which to some extent limits its further clinical application. Steady-state flash visual evoked potentials (SSFVEP) is a type of visual electrophysiological response that records corresponding evoked potentials by flashing stimuli at a fixed frequency on the eyes. SSFVEP uses a high-frequency flashing stimulus that produces an environment similar to light adaptation, resulting in the inhibition of rod cell electrical activity. Therefore, it can be considered as directly reflecting the activity of the incoming nerve fibers connected to cone cells, thus better reflecting the integrity of the neural pathway from the macular cones to the cortical visual area^9^.

In our present study, we utilized SSFVEP to evaluate the visual function of patients with concurrent opacities due to the ocular media (vitreous hemorrhage). Besides, we also compared the sensitivity of the P2-wave amplitude from FVEP and the average amplitude of SSFVEP in the same diseased eyes and those without vitreous hemorrhage. We aimed to assess the role and sensitivity of SSFVEP in the evaluation of ocular fundus disease, like vitreous hemorrhage. Our data revealed that no significant difference in the waveform of FVEP exited between both eyes, while the waveform of SSFVEP the diseased eye was dramatically flattened. Furthermore, we found that the average amplitude of SSFVEP in the diseased eye was statistically lower than that without vitreous hemorrhage, while the amplitude of FVEP was not obviously changed. In addition, the difference ratio of SSFVEP amplitude between both eyes was significantly greater than that of FVEP amplitude. Together, our results indicated that SSVEP had a relatively higher sensitivity in evaluating visual function from the same eyes with vitreous hemorrhage compared to FVEP.

As far as we know, there were only a few clinical studies on the application of SSFVEP in ocular diseases. SSFVEP induced by 10 Hz flashing stimuli was reported to show some values in assessing the visual function after cataract surgery. Weinstein et al.^11^ suggested that preoperative SSFVEP might be used to predict the visual function of patients after removing opacities (cataracts). Similar application of SSFVEP was reported by other researchers^18^. In addition, a single-eye SSFVEP induced by 10 Hz flashing stimuli in 44 patients with diabetic retinopathy was analyzed in another study^19^. The study found that when SSFVEP was assessed as abnormal, the final postoperative visual acuity of the patients was lower (100%). Furthermore, the worse the SSFVEP abnormality level, the worse the postoperative visual acuity. Additionally, Wu et al. reported the application of SSFVEP stimulated by 30 Hz flash to detect characteristic values of various types of optic neuropathy, and found that SSFVEP waveforms were abnormal in patients with optic neuritis, retrobulbar neuritis, ischemic optic neuropathy, optic nerve contusion, and optic atrophy^20^. These research results were consistent with ours, which might suggest SSFVEP as a novel potential application for visual function assessment.

There were some limitations of our study. Firstly, the stimulation frequency for SSFVEP has not yet been unified clinically. Previous studies used 10 Hz or 30 Hz, while our study used 12 Hz. Secondly, the sample size of patients included in our study was relatively small due to the COVID-19 pandemic when our study was conducted. Additionally, we did not distinguish the difference of SSFVEP for different fundus diseases, such as diabetic retinopathy, retinal vein occlusion and polypoidal choroidal vasculopathy.

In summary, our preliminary clinical findings suggest that SSFVEP had a higher sensitivity than FVEP in some fundus diseases with vitreous hemorrhage, providing a novel potential application for visual function assessment. Further research with a larger sample size, different stimulation frequencies, and different ocular fundus diseases is warranted to comprehensively assess and validate the application of SSFVEP in evaluating visual function of ocular diseases.

## Data Availability

The datasets used and/or analysed during the current study available from the corresponding author on reasonable request.
